# Müller cell degeneration and microglial dysfunction in the Alzheimer’s retina

**DOI:** 10.1186/s40478-022-01448-y

**Published:** 2022-10-05

**Authors:** Qinyuan Alis Xu, Pierre Boerkoel, Veronica Hirsch-Reinshagen, Ian R. Mackenzie, Ging-Yuek Robin Hsiung, Geoffrey Charm, Elliott F. To, Alice Q. Liu, Katerina Schwab, Kailun Jiang, Marinko Sarunic, Mirza Faisal Beg, Wellington Pham, Jing Cui, Eleanor To, Sieun Lee, Joanne A. Matsubara

**Affiliations:** 1 MD Undergraduate Program, University of British Columbia, Vancouver, BC Canada; 2 Postgraduate Medical Education, Department of Family and Community Medicine, University of Toronto, Barrie, ON Canada; 3 School of Engineering Sciences, Simon Fraser University, Burnaby, BC Canada; 4 Mental Health & Clinical Neurosciences, School of Medicine, University of Nottingham, University of Nottingham, Nottingham, England; 5 Department of Pathology and Laboratory Medicine, University of British Columbia, Vancouver, BC Canada; 6 Division of Neurology, Department of Medicine, University of British Columbia, Vancouver, BC Canada; 7 Department of Ophthalmology and Visual Sciences, Eye Care Centre, University of British Columbia, 2550 Willow Street, Vancouver, BC V5Z3N9 Canada; 8 Vanderbilt University Institute of Imaging Science, Vanderbilt University School of Medicine, Nashville, USA

**Keywords:** Retina, Amyloid-β, Alzheimer’s disease, Biomarker, Macroglia, Microglia

## Abstract

Amyloid beta (Aβ) deposits in the retina of the Alzheimer’s disease (AD) eye may provide a useful diagnostic biomarker for AD. This study focused on the relationship of Aβ with macroglia and microglia, as these glial cells are hypothesized to play important roles in homeostasis and clearance of Aβ in the AD retina. Significantly higher Aβ load was found in AD compared to controls, and specifically in the mid-peripheral region. AD retina showed significantly less immunoreactivity against glial fibrillary acidic protein (GFAP) and glutamine synthetase (GS) compared to control eyes. Immunoreactivity against ionized calcium binding adapter molecule-1 (IBA-1), a microglial marker, demonstrated a higher level of microgliosis in AD compared to control retina. Within AD retina, more IBA-1 immunoreactivity was present in the mid-peripheral retina, which contained more Aβ than the central AD retina. GFAP co-localized rarely with Aβ, while IBA-1 co-localized with Aβ in more layers of control than AD donor retina. These results suggest that dysfunction of the Müller and microglial cells may be key features of the AD retina.

**Supplementary Information:**

The online version contains supplementary material available at 10.1186/s40478-022-01448-y.

## Introduction

Dementia is a multifactorial cognitive disorder, impacting memory, daily activities, and communication leading to significant disability and dependency in the elderly. Worldwide, 47 million people, or 5% of the global geriatric population, are affected, with an estimated annual cost of $818 billion dollars [87]. The prevalence and impact establish dementia as a public health and research priority. Alzheimer’s disease (AD), characterized by the formation of plaques and neurofibrillary tangles in the brain, is the most common form of dementia and constitutes ~ 70% of all dementias. Although AD was first described more than 100 years ago, even current cutting edge therapeutics such as Donanemab have failed to impact secondary outcomes such as cognition and severity of dementia, despite slowed decline measured by the Integrated Alzheimer’s Disease Rating Scale [2, 43, 54]. The consistent failure to treat AD through the targeting of amyloid plaques has indicated that they may not be suitable targets for treating AD, or that anti-amyloid treatment methods necessitates a sensitive and precise method for staging early AD [43]. Clearly more information is needed to understand AD, especially the earliest stages of AD, to prevent its development.

Pathological hallmarks of AD in the brain include senile or neuritic plaques containing extracellular amyloid-β (Aβ) and neurofibrillary tangles composed of hyper-phosphorylated tau proteins (pTau) [47]. Aβ is an attractive brain biomarker for the early detection of AD, as it may accumulate up to 20 years prior to clinical presentation of dementia [3, 30]. However, conventional structural imaging of the brain such as CT or MRI is often not sensitive enough to detect the subtle changes associated with early AD pathology [33, 34]. Other methods to measure Aβ, such as in blood or cerebrospinal fluid are currently under development. Positron emission tomography (PET) with C^11^ labelled Pittsburgh compound B has been shown to distinguish Aβ levels in AD and non-AD brain scans, but this method is costly, invasive and not feasibly deployed in community settings [59].

Recently, there has been a focus on the retina as an alternative tissue bed to assess Aβ load in AD. Unlike the brain, the retina can be readily examined in vivo using noninvasive, light-based imaging techniques such as fluorescent scanning laser ophthalmoscopy and optical coherence tomography (OCT). Examination of the retina for Aβ load has already attracted significant interest, as it is more accessible and cost-effective than neuroimaging of the central nervous system (CNS) [11–13, 37, 50, 53]. Our group and others demonstrated that Aβ is deposited in the AD retina, thus identifying the retina as a surrogate tissue in which to assess Aβ-associated pathology [37, 42]. In addition to Aβ deposits, the AD retina displays other AD-related pathological changes including degeneration of retinal ganglion cells [4, 5, 27], significant loss and abnormal morphology of melanopsin retinal ganglion cells [40], changes in vasculature, overall thinning of the retinal nerve fiber layer (RNFL), ganglion cell layer (GCL), and choroidal layers [23, 40, 52] further emphasizing the utility of imaging the retina for AD biomarkers.

In the mammalian eye, macroglial and microglial cells live in a symbiotic relationship with retinal neurons. Retinal macroglia includes astrocytes and Müller cells [9, 56]. Astrocytes have flattened cell bodies with radiating processes that are brightly stained by dyes binding to glial fibrillary acidic protein (GFAP) [36, 71]. They are seen almost entirely in the RNFL [56, 71]. Astrocytes envelop neuronal axons and blood vessels to aid in the homeostasis of the neuroretina [56]. Müller cells, on the other hand, span the entire width of the neuroretina from outer limiting membrane to inner limiting membrane [63]. They function to support neuronal metabolic function through mechanisms such as metabolizing neurotransmitters such as glutamate that are secreted by neurons [16, 65]. As a result, Müller cells can be identified by glutamine synthetase (GS) immunoreactivity [48, 66]. When activated by retinal insult, Müller cells also express GFAP [51, 70]. Microglial cells are found in every layer of the retina [56]. They perform phagic function after trauma and can be visualized using an antibody against ionized calcium binding adapter molecule-1 (IBA-1) [32, 56, 57].

Given that the AD retina displays pathological features of neurodegeneration, we hypothesized that retinal glial cells play a role in these neurodegenerative events by responding to the increased Aβ deposits in the AD eye. The current study investigates the laminar distribution and the spatial relationships between Aβ, macroglia and microglial cell populations in the AD retina. A better understanding of glial populations and neurodegenerative events associated with Aβ in the AD eye will inform and prioritize the most relevant features to assess by in vivo ophthalmic imaging towards early and accurate detection and staging of AD.

## Materials and methods

### Neuropathological assessment of Alzheimer’s disease

Neuropathological diagnoses of donor brain tissues were provided by clinical neuropathologists according to National Institute on Aging Alzheimer’s Association guidelines for the neuropathologic assessment of Alzheimer’s disease [31]. Diagnostic data are provided in Table 1. Post-mortem brain and retinal tissues from donors with AD (N = 9) were obtained from the Department of Pathology and Laboratory Medicine at Vancouver General Hospital (VGH). Post-mortem retinal tissues from control eyes (age-matched controls without dementia) were obtained from the Eye Bank of British Columbia (N = 12). Exclusion criteria for control eyes included central nervous system disorders including AD, multiple sclerosis, Parkinson’s disease, and amyotrophic lateral sclerosis. The AD and control eyes were obtained from donors whose ages ranged between 55 and 89 years. The mean age of AD and control donors was 77.3 years and 74.8 years, respectively. There was no significant difference between the mean ages of the two groups (Mann–Whitney, *p* = 0.12).

**Table 1:** Demographics and neuropathological assessment of AD donor brains

Donor ID	Age	Sex	Primary path Dx	Additional Path Dx	A-beta (Thal) (1–5)	Braak stage (1–6)	Neuritic plaque (CERAD, Biel)	Diffuse plaque (CERAD, Biel)	Preparation
A1	89	M	AD	LBD, TDP, HS, CVD, CAA	5	6	Frequent	Frequent	Cross section
A2	82	F	AD	CAA	5	6	Frequent	Frequent	Cross section
A3	55	F	AD	N/A	5	6	Frequent	Sparse	Cross section
A4	84	M	AD, CVD	N/A	5	6	Frequent	Moderate	Cross section
A5	83	M	DLB	Mild AD	3	3	Sparse	Sparse	Cross section
A6	70	M	AD	CAA	5	6	Moderate	Frequent	Punch
A7	80	M	DLB	Moderate AD	3	4	Moderate	Frequent	Punch
A8	76	F	AD	Mild HS	5	6	Frequent	Frequent	Punch
A9	80	M	AD	CAA, CVD	5	6	Frequent	Frequent	Cross section
C1	80	M	Control	Control	N/A	N/A	N/A	N/A	Cross section
C2	80	N/A	Control	Control	N/A	N/A	N/A	N/A	Cross section
C3	72	F	Control	Control	N/A	N/A	N/A	N/A	Cross section
C4	75	M	Control	Control	N/A	N/A	N/A	N/A	Cross section
C5	75	M	Control	Control	N/A	N/A	N/A	N/A	Cross section
C6	79	M	Control	Control	N/A	N/A	N/A	N/A	Cross section
C7	75	M	Control	Control	N/A	N/A	N/A	N/A	Cross section
C8	70	M	Control	Control	N/A	N/A	N/A	N/A	Punch
C9	68	M	Control	Control	N/A	N/A	N/A	N/A	Punch
C10	74	M	Control	Control	N/A	N/A	N/A	N/A	Punch
C11	N/A	N/A	Control	Control	N/A	N/A	N/A	N/A	Cross section
C12	N/A	N/A	Control	Control	N/A	N/A	N/A	N/A	Cross section

*AD* Alzheimer’s disease; *DLB* Dementia with Lewy bodies; *CVD* Cerebrovascular dementia; *FTLD-TDP* Frontotemporal lobar degeneration with TDP-43 inclusions; *CAA* Cerebral amyloid angiopathy; *HS* Huntington’s disease; *N/A* Not applicable (Control eyes). “A” stands for Alzheimer Disease eyes. “C” stands for control eyes. “N/A” stands for not available, or unknown. AD donors mean age was 77.4 (N = 9). Controls mean age was 74.8 (N = 12). There was no significant difference between the mean ages of the two groups (Mann–Whitney, *p* = 0.12)

Retinal samples were processed as paraffin embedded cross-sections. (5 µm thickness, N = 15) or free-floating punches (4 mm, N = 6). Only one eye per donor was processed for this study. Slides with cross-sections of eye tissues were deparaffinized and rehydrated through a series of xylene rinses followed by rinses in decreasing percentages of alcohol solutions to distilled water. Slides were then washed in triplicate in Phosphate Buffered Saline (PBS) pH 7.2 for 5 min each. Methods for processing retinal punches followed the procedures in our earlier study [42].

### Immunohistochemistry for TUBB3, GFAP or IBA-1 and double labelling with BA4

Antigen retrieval of Aβ was undertaken by incubation of slides in 88% formic acid for 5 min at room temperature. In separate cohorts, Tubulin β 3 class III (TUBB3), GFAP and IBA-1 antigens were retrieved with 10 mM sodium citrate, 0.05% Tween 20, pH 6.0 at 100 °C for 10 to 20 min. Tissues were then washed in PBS pH 7.2 for 5 min three times. Next, sections were blocked with normal serum by incubating in 3% normal goat serum and 0.3% TX-100 PBS for 20 min at room temperature.

Aβ immunohistochemistry employed the monoclonal mouse antibody against human β-amyloid, BA4 (clone 6F/3D -Agilent, CA, USA), which labels β-amyloid containing the N-terminal epitope, consisting of residues 8–17 (ser-gly-tyr-glu-val-his-his-gln-lys-leu) of Aβ which are the same as residues 660–669 of amyloid precursor protein (APP), and therefore theoretically may label APP. However, we also used two additional primary antibodies against human β-amyloid. Immunostaining patterns using BA4 were identical to those of 12F4 in our study (Fig. 2). 12F4 is an antibody specific to Aβ according to the literature ([49], see Table 1), which labels the C-terminus of β-amyloid and is specific for the isoform ending at the 42nd amino acid (Biolegend, CA, USA). The third antibody, 6E10, recognizes the epitope that lies within the amino acids 3–8 of β-amyloid as well as the precursor forms (Biolegend, CA, USA) (Table 2). Primary antibodies were diluted in 3% normal goat serum and 0.3% TX100 PBS. Sections were incubated at room temperature for 2 h before incubation at 4 °C overnight.

**Table 2:** List of antibodies used

Antigen	Antibody (catalog no.)	Dilution	Source
*Primary antibodies*			
Aβ	Monoclonal Mouse Anti-Human Beta-Amyloid Clone 6F/3D (M0872)	1:100	Agilent/Dako
TUBB3	Purified rabbit anti-Tubulin P 3 (845501)	1:500	Biolegend
GFAP	Polyclonal rabbit anti-Glial Fibrillary Acidic Protein antibody (Z0334)	1:300	Agilent/Dako
GS	Purified Monoclonal Mouse Anti-Glutamine Synthetase Antibody, clone GS-6	1:300	MilliporeSigma
IBA-1	Polyclonal rabbit Anti-IBA-1 antibody (019–19741)	1:500	Wako
12F4	Purified mouse Anti-β- Amyloid, 1–42 Clone 12F4 (805504)	1:100	Biolegend
6E10	Purified mouse Anti-β-Amyloid, 1–16 Clone 6E10 (803001)	1:100	Biolegend
*Secondary antibodies*			
Cy3	Goat Anti-mouse Cy3 Alexa 546 IgG1 secondary antibody (A21123)	1:400	Fisher scientific
FITC	Goat Anti-rabbit Alexa 488 secondary antibody (A11070)	1:500	Fisher scientific

In separate double labelling cohorts, TUBB3, GFAP, or IBA-1 primary antibody incubation followed incubation in the primary antibody against Aβ. A dilution of purified rabbit anti-rat brain anti-Tubulin β 3 class III (TUBB3), polyclonal rabbit anti-Glial Fibrillary Acidic Protein (GFAP) antibody, or polyclonal rabbit anti-Ionized calcium binding adapter molecule-1 (IBA-1) antibody was made in 3% normal goat serum and 0.3% TX100 PBS (Table 2). Sections were incubated at room temperature for 1 h before incubation at 4 °C overnight. Tissues were then washed in PBS pH 7.2 for 5 min three times.

Secondary antibody incubation was performed in the following fashion. Sections were incubated in Cy3 Alexa-labelled goat anti mouse secondary antibody at room temperature for 45 min to visualize Aβ resulting in red (543 nm) fluorescence (Table 2). Sections were then sequentially incubated in Goat Anti-rabbit Alexa 488 secondary antibody at room temperature for 45 min to visualize TUBB3, GFAP or IBA-1 in different experimental cohorts, resulting in green (488 nm) fluorescence (Table 2). After secondary antibody incubation, slides were then washed in PBS pH 7.2 for 5 min three times.

Nuclear staining was performed using 1:500 DAPI in PBS incubation at room temperature for 10 min. Tissues were then washed in PBS pH 7.2 for 15 min four times. Finally, slides were coverslipped using glycerol and PBS (80:20) and #1.5 coverslips and sealed by enamel. Slides were stored at 4 °C and protected from light between confocal imaging sessions. Negative control slides were prepared by omission of the primary antibody, with all subsequent steps identical and in parallel with the slide processing.

### Immunohistochemistry for GFAP and double labelling with GS

Antigen retrieval of GS was undertaken by incubation of slides in diluted Proteinase K in Tris–EDTA buffer for 10 min at room temperature. Tissues were then washed in PBS pH 7.2 for 5 min three times. Next, sections were blocked with normal serum by incubating in 3% normal goat serum and 0.3% TX-100 PBS for 20 min at room temperature.

GFAP immunohistochemistry employed the polyclonal rabbit anti-Glial Fibrillary Acidic Protein (GFAP) antibody (Agilent/Dako) (Table 2). Primary antibodies were diluted in 3% normal goat serum and 0.3% TX100 PBS. Sections were incubated at room temperature for 1 h before incubation at 4 °C overnight. For secondary antibody immunostaining, sections were incubated in Goat Anti-rabbit Alexa 488 secondary antibody at room temperature for 45 min to visualize GFAP resulting in green (488 nm) fluorescence (Table 2). Slides were then washed in PBS pH 7.2 for 5 min three times.

GS primary antibody incubation followed incubation in the primary antibody against GFAP. A dilution of purified monoclonal mouse anti-Glutamine Synthetase antibody, clone GS-6 (GS) antibody (MilliporeSigma) was made in 3% normal goat serum and 0.3% TX100 PBS (Table 2). Sections were incubated at room temperature for 1 h before incubation at 4 °C overnight. Tissues were then incubated at room temperature for 2 h before washed in PBS pH 7.2 for 5 min three times. For secondary antibody immunostaining, sections were incubated in Cy3 Alexa-labelled goat anti mouse secondary antibody at room temperature for 45 min to visualize GS resulting in red (543 nm) fluorescence (Table 2). Slides were then washed in PBS pH 7.2 for 5 min three times.

Nuclear staining was performed using 1:500 DAPI in PBS incubation at room temperature for 10 min. Tissues were then washed in PBS pH 7.2 for 15 min four times. Finally, slides were coverslipped using glycerol and PBS (80:20) and #1.5 coverslips and sealed by enamel. Slides were stored at 4 °C and protected from light between confocal imaging sessions. Negative control slides were prepared by omission of the primary antibody, with all subsequent steps identical and in parallel with the slide processing.

### Terminology

**Fig. 1:**
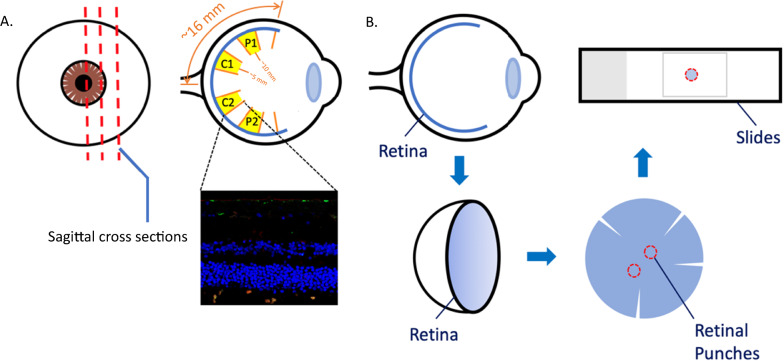
Post-mortem human retina preparation. **A** Paraffin embedded sagittal cross-sections. (5 μm in thickness). The retina was divided into 4 sectors. Central retina, C1 and C2, were sectors adjacent to the optic nerve head, around 5 mm away measured circumferentially. Sectors P1 and P2 were mid-peripheral retinal zones, around 10 mm away from the optic nerve head measured circumferentially. **B** Preparation of free-floating retinal punches and wholemount (4 mm in diameter). Schematic not to scale

Retinal regions refer to whether the retina image is taken from central (~ 5 mm from optic nerve head or ONH, measured circumferentially) or mid-peripheral (~ 10 mm from Optic Nerve Head or ONH, measured circumferentially) retina (Fig. 1). Cellular profiles refer to the four types of cell markers used for labelling with TUBB3 (neurons), GFAP (macroglia consisting of astrocytes and Müller cells), GS (Müller cells) and IBA-1 (microglia). In the following sections, TUBB3, GFAP, and IBA-1 cohorts refer to the samples double labelled with the respective markers and BA4 (Sect. 2.3). GS cohort refers to the samples double labelled with GS and GFAP (Sect. 2.4). Donor eye tissues were processed in cross sections in which all layers of the retina are visible, or in wholemount punch preparations in which the full thickness of the retina is intact and layers are visualized by confocal optical imaging using depth (z-) stacks. Retinal layers were arbitrarily grouped into inner layers (RNFL, GCL, IPL) and outer layers (INL, OPL, ONL), to facilitate the analysis and reporting of results.

### Confocal microscopy and autofluorescence

After immunostaining, retinal sections were imaged using a Zeiss 800 confocal microscope with Zen 2.6 Blue version software. Aβ labelling by Cy3 was imaged at 543 nm. TUBB3, GFAP, or IBA-1 labelling by FITC was imaged at 488 nm. Nuclear labelling by DAPI was imaged with 405 nm. In a separate cohort, GS and GFAP double labelled slides were imaged at 543 nm to visualize GS and 488 nm to visualize GFAP.

**Fig. 2:**
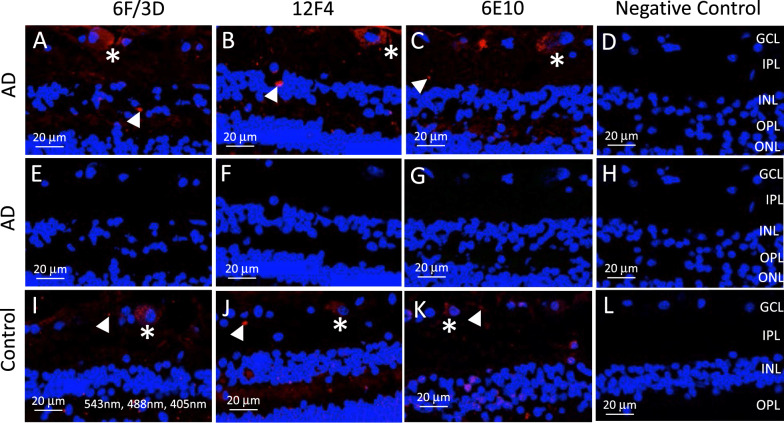
Comparisons of immunofluorescence using three Aβ antibodies. Cross sections of Alzheimer’s disease (AD) retina were processed with three monoclonal mouse antibodies against Aβ: **A**, **E** Clone 6F/3D, which labels β-amyloid containing the N-terminal epitope (Agilent, CA, USA); **B**, **F** 12F4, which labels the C-terminus of β-amyloid and is specific for the isoform ending at the 42nd amino acid (Biolegend, CA, USA); and **C**, **G** 6E10, which recognizes the epitope that lies within the amino acids 3–8 of β-amyloid as well as the precursor forms (Biolegend, CA, USA). Note that immunolabeling pattern is consistent with all three antibodies and identifies what is likely intracellular labelling of retinal ganglion cells (asterisks) and extracellular deposits (arrowheads). Negative control sections in which the primary antibody was omitted resulted in no immunofluorescence (**D**). All sections were also imaged under 488 nm for autofluorescence that may be associated with melanopsin containing retinal ganglion cells. Note that no green or yellow/orange signals were observed in **A**–**D** in which both 543 nm and 488 nm were used, and confirmed in **E**–**H** in which the same section was imaged under 488 nm only. **I-L**) Cross sections of control retina were also processed for all three monoclonal mouse antibodies against Aβ and demonstrated both what is likely intracellular labelling of retinal ganglion cells (asterisks) and extracellular deposits (arrowheads). DAPI was used to label nuclei throughout all panels and imaged under 405 nm

It is known that melanopsin containing retinal ganglion cells exhibit autofluorescence, which may contaminate the analysis used in this study to quantify Aβ immunofluorescence. To differentiate potential autofluorescence (due to melanopsin) from Aβ immunofluorescence, we imaged Aβ under 543 nm (wavelength specific for the secondary antibody against Aβ) and 488 nm (wavelength used to identify autofluorescence). Figure 2A-H demonstrates AD retina after Aβ immunofluorescence using 6F/3D, 12F4 or 6E10 antibodies against Aβ and imaged under 543 nm and 488 nm (Fig. 2A–D) or under 488 nm alone (Fig. 2E–H). Note that only the red immunofluorescence (associated with Aβ) was visible within what are likely to be retinal ganglion cells (asterisks) and extracellular deposits (arrowheads). When the same areas were imaged under 488 nm alone (wavelength associated with autofluorescence), none of the ganglion cells demonstrated autofluorescence (Fig. 2E–H). A previous study showed that melanopsin containing retinal ganglion cells in the human retina represent a very sparse cell population, comprising only 0.4% of the 1.07 million ganglion cells in the human retina [14, 46]. As we did not observe autofluorescence of the retinal ganglion cells and supported by the literature that melanopsin containing retinal ganglion cells only comprise 0.4% of the retinal ganglion population, we concluded that the quantification of Aβ by immunofluorescence was not significantly affected, if at all, by melanopsin autofluorescence.

## Quantitative analysis

### Data

Quantitative analysis of the immunostaining was performed using confocal images of the cross-sectional samples. Two cross-sections were analyzed from each donor, and in each cross-section two images from the central region (~ 5 mm from ONH) and two images from the mid-peripheral region (~ 10 mm from ONH), determined by their distance from the optic nerve head, were taken (Fig. 1A). A total of 81 images from the TUBB3 cohort, 96 images from the GFAP cohort, 91 images from the IBA-1 cohort, and 68 images from the GS cohort were obtained. Difference in the image numbers between the cohorts is due to loss of intact retinal tissue during processing, or low imaging quality. Each image was examined for image and tissue quality, and to identify immunostaining artifacts due to uneven sectioning, edge artifacts or small tears in retina tissue. If an artifact was identified within a particular layer, the data point from the layer was excluded from analysis, allowing for preservation of the data from the other layers in the image. Some images were completely excluded from the analysis because the tissue was damaged during processing and the retinal layers were not readily segmentable. In each cohort, a unique data point was defined by donor and region (central (~ 5 mm from ONH), mid-peripheral (~ 10 mm from ONH)) in each layer.

#### Processing

In each cross-section image, retinal nerve fiber layer (RNFL), ganglion cell layer (GCL), inner plexiform layer (IPL), inner nuclear layer (INL), outer plexiform layer (OPL), and outer nuclear layer (ONL) boundaries were manually segmented using ITK-SNAP [89]. The images were first exported from the native.czi format to.tiff format using Zeiss Zen software, and the seven boundaries separating the six retinal layers were segmented in ITK-SNAP [88]. Using the manual delineations, each pixel in the image was labelled as belonging to one of the retinal layers or the background. In the TUBB3, GFAP, and IBA-1 cohorts, each pixel was labelled by the presence of immunostaining using intensity thresholding in the red channel for Aβ positivity, and in the green channel for TUBB3, GFAP or IBA-1 positivity. In the GS cohort, each pixel was labelled in the red channel for GS positivity and in the green channel for GFAP positivity. In order to empirically choose the intensity thresholds, multiple threshold values were obtained on a set of test images. These were then assessed by two blinded raters (ET and JAM), who selected the optimal threshold value by comparing the threshold-masked images with their original images. Based on the pixel labelling, the layer-wise percentages Aβ/TUBB3/GFAP/IBA-1/GS positive pixels, normalized by the total number of pixels in the layer were calculated.

Co-localization of Aβ with TUBB3/GFAP/IBA-1 was examined for two hypotheses: (i) whether Aβ deposition occurred preferentially at the locations of neurons/macroglia/microglia for both AD and control groups, and (ii) Aβ co-localization with microglia is different between the two groups, suggesting a difference in the ability of microglia to phagocytose Aβ. To test the first hypothesis, we compared the percentages of Aβ co-localization in the TUBB/GFAP/IBA-1 positive region vs. TUBB/GFAP/IBA-1 negative region in each image. If there was no co-localization, then these two percentages should not differ, which would infer that Aβ was randomly distributed. To test the second hypothesis, we compared the percentage of Aβ deposition that was co-localized by microglia between the control and AD groups.

Co-localization of GS with GFAP was examined for two hypotheses: (i) GFAP labelled both astrocytes and Müller cells whereas GS labelled only Müller cells, and (ii) astrocytes and Müller cells display varying abundance in AD vs control retina. To test these hypotheses, we compared the AD and control groups separately for the pixels that were labelled by both GS and GFAP, GS-only, and GFAP-only.

The image processing and parameter computation were performed using MATLAB (The MathWorks Inc., Natick, USA).

### Statistical analysis

Parameters were grouped by layer (RNFL, GCL, IPL, INL, OPL, ONL) and group (control, AD). In each group (control, AD), parameter values were averaged for each layer. The group averages of the control and AD retinas were compared layer-wise by nonparametric Wilcoxon test. Outliers were detected and excluded from each group-layer average using a sample-size adapted method [29]. Following removal of outliers, the results were plotted. Statistical analysis and visualization were performed using R [81]. All p-values can be found in Additional file 2.

## Results

### Retinal Aβ load is higher in AD compared to control eyes

**Fig. 3:**
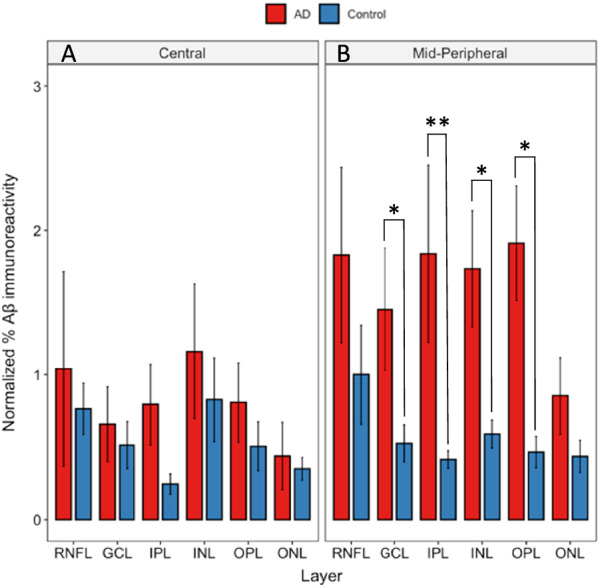
Layer-wise retina Aβ load in Alzheimer’s disease (AD) retina compared to controls. Red bars represent AD donors (N = 5). Blue bars represent age-matched controls (N = 7). BA4 labelled post-mortem human retina cross-sections were imaged at central and mid-peripheral locations in relation to optic nerve head. Normalized area percentage of BA4 labelled Aβ was calculated in each retinal layer and plotted against the particular layer. Aβ was significantly higher in the AD donors in the mid-peripheral GCL (*p* < 0.05), IPL (*p* < 0.01), INL (*p* < 0.05), and OPL (p < 0.05). However, in central retina there was no significant differences between AD and control retina. * represent *p* < 0.05. ** represent *p* < 0.01. Error bars = Standard Error

Figure 3 displays layer-wise axial profile of Aβ deposition in the post-mortem human retina. The percentage of Aβ/BA4 positive pixels of a particular layer (shown on the y-axis) was plotted against individual retinal layers (shown on the x-axis) in the central (~ 5 mm from ONH) (A) and mid-peripheral (~ 10 mm from ONH) (B) retina. The percentage of Aβ positive pixels was found to be significantly higher in the mid-peripheral (~ 10 mm from ONH) retina of the AD donors compared to controls, specifically in the GCL (*p* < 0.05), IPL (*p* < 0.01), INL (*p* < 0.05), and OPL (*p* < 0.05) (Fig. 3). Interestingly, no significant difference was observed between AD and controls in the central retina (~ 5 mm from ONH). These results were consistent with our earlier findings in which we assessed Aβ deposition in retinal wholemounts [42].

### Less macroglia and more microglial immunoreactivity found in AD compared to control eyes

**Fig. 4:**
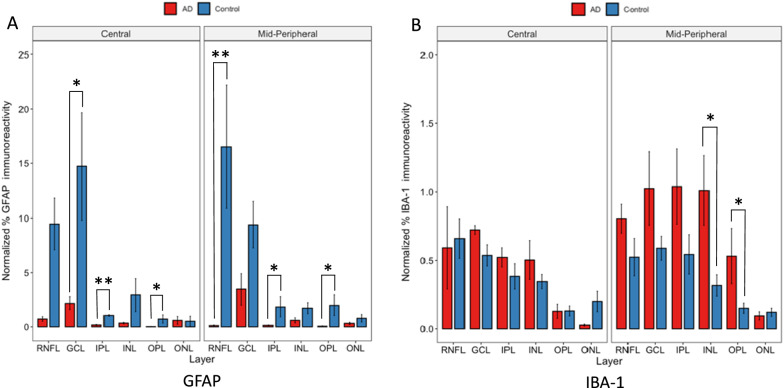
Layer-wise GFAP and IBA-1 in Alzheimer’s disease (AD) retina compared to controls. Red bars represent AD donors (N = 5). Blue bars represent age-matched controls (N = 7). **A** GFAP labelled post-mortem human retina cross-sections are imaged at central and mid-peripheral locations in relation to optic nerve head. Normalized area percentage of GFAP positive pixels is calculated in each retinal layer and plotted against the particular layer. GFAP immunoreactivity, is **lower** in AD donors, as seen by the red bars generally lower than the blue bars. This is significant in the central GCL (*p* < 0.05), IPL (*p* < 0.01), and OPL (*p* < 0.05) and mid-peripheral RNFL (*p* < 0.01), IPL (*p* < 0.05), and OPL (*p* < 0.05). **B** IBA-1 labelled post-mortem human retina cross-sections are imaged at central and mid-peripheral locations in relation to optic nerve head. Normalized area percentage of IBA-1 positive pixels is calculated in each retinal layer and plotted against the particular layer. IBA-1 immunoreactivity is **higher** in AD donors, as seen by the red bars generally higher than the blue bars. This is significant in mid-peripheral INL and OPL (*p* < 0.05). Note that the ranges of the y-axes of the two panels are different as there was generally more GFAP immunoreactivity than IBA-1 throughout our study * represent *p* < 0.05. **represent *p* < 0.01. Error bars = Standard Error

To reveal glial distribution in the AD retina, we studied macroglia and microglia in each retinal layer. Macroglia labelling was measured by the percentage of GFAP-positive pixels, while microglial labelling was measured by the percentage of IBA-1-positive pixels in each retinal layer. These percentages of GFAP or IBA-1 positive pixels were plotted against individual retinal layers (Fig. 4).

In the central retina (~ 5 mm from ONH), both AD and control eyes displayed similar levels of IBA-1 immunoreactivity, while in the mid-peripheral retina (~ 10 mm from ONH), the AD eye (red bars) displayed higher levels of IBA-1 compared to the control eye (blue bars). Significance was reached in mid-peripheral retina only (~ 10 mm from ONH), and in one (INL) of the three inner layers and one (OPL) of the three outer layers.

### Müller marker shows a significant decline in AD compared to controls in mid-peripheral retina

**Fig. 5:**
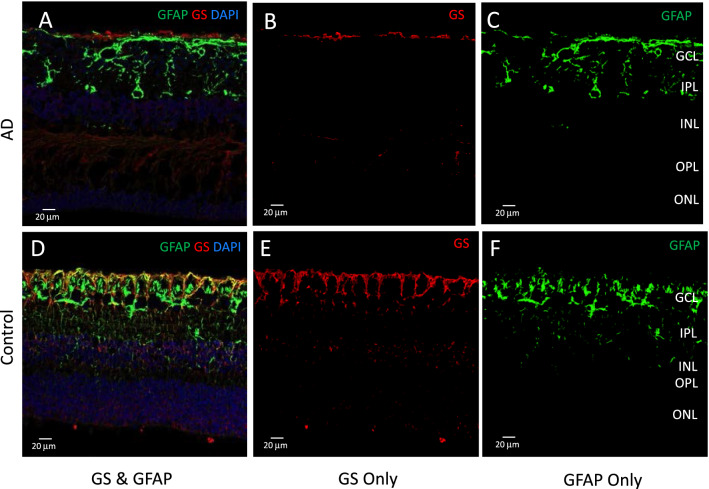
Thresholding for GS and GFAP immunostaining. Glutamine synthetase (GS) immunolabelling (red) marks both resting and activated Müller cells. Glial filamentary acidic protein (GFAP) immunolabelling (green) is present in activated Müller cells and astrocytes. Representative images of cross-sections stained with GS and GFAP. **A, D** Pixels positive for both GS and GFAP (yellow) activated Müller cells. **B, E** Pixels positive for GS Only represent resting Müller cells. **C, F** Pixels positive for GFAP Only represent astrocytes

**Fig. 6:**
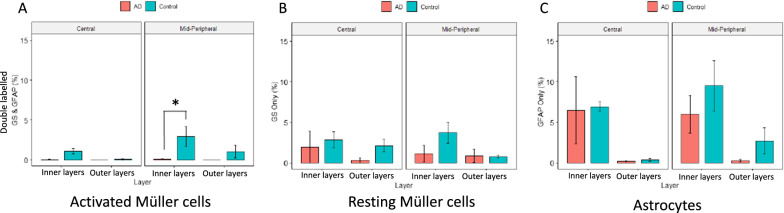
Quantitative analysis of GS and/or GFAP staining in AD compared to control eyes. Retinal layers were grouped into inner layers (RNFL, GCL, IPL) and outer layers (INL, OPL, ONL). Red bars represent AD eyes, green bars represent control eyes. **A** Percentage of pixels double labelled by both GS & GFAP in inner and outer retina is shown. Activated Müller cells, labelled with both GS & GFAP, shows lower levels of immunoreactivity in AD (red bars) compared to control eyes (green bars). This was significant in the inner layers of mid-peripheral retina. **B** Percentage of pixels labelled with GS only in inner and outer retina is shown. **C** Percentage of pixels labelled with GFAP only in inner and outer retina is shown. * represent *p* < 0.05. Error bars = Standard Error

GFAP labels macroglia in the retina, which includes astrocytes and Müller cells [70]. While resting Müller cells express minimal levels of GFAP, activated Müller cells display upregulation of GFAP expression after retinal injury [20, 21, 44, 51, 70]. Since GFAP alone cannot be used to distinguish between astrocytes and Müller cells, GS immunolabelling was performed to better understand the role of the Müller cell, a specialized macroglial cell only found in the retina [20]. Confocal images were taken for GFAP and GS labelled post-mortem human retinal cross-sections, and in separate red and green channels the images were segmented pixel-wise into GS (red)—or GFAP (green)—positive areas. Figure 5 A and D shows a representative confocal image with the red, green and blue channels. Pixels positive for both GS & GFAP represent activated Müller cells, as only activated (not resting) Müller cells express GFAP. Figure 5B and E show an example confocal image derived by subtracting GS & GFAP double labelled pixels from all GS pixels, yielding the GS only pixels with no GFAP staining, representing resting Müller cells. Figure 5C and F show an example confocal image derived by subtracting GS & GFAP double labelled pixels from all GFAP pixels, yielding the GFAP only pixels with no GS staining, representing astrocytes. In each image, the amount of GS and/or GFAP staining in the inner layers (NFL, GCL, IPL) and outer layers (INL, OPL, ONL) was calculated as the percentage of the positive pixels in each region, and the group averages were compared between the AD and control groups, as shown in Fig. 6. Activated Müller cells (GS & GFAP) shows lower levels of immunoreactivity in AD (red bars) compared to control eyes (green bars). This was significant in the inner layers of mid-peripheral retina (~ 10 mm from ONH) (Fig. 6A). Astrocytes (without the Müller cell component, *i.e.,* GFAP only) show similar immunoreactivity for AD (red bars) and control eyes (green bar) (Fig. 6C). This suggests that amongst the GFAP positive cells within the retina, it is the Müller cell that demonstrates lower immunoreactivity in AD eyes compared to age-matched control eyes.

### Representative images for Aβ with neuronal and glial cell markers in the AD and control eyes

**Fig. 7:**
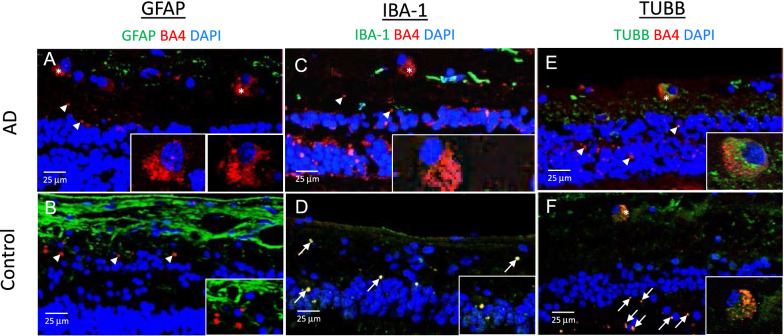
Representative immunofluorescence images of retinal cross-sections. **A**, **C, E** Post-mortem human retina samples were processed from AD donors (Mean age = 78.6) (N = 5). **B**, **D**, **F** Post-mortem human retina samples were processed from controls (Mean age = 76.8) (N = 6). Tissues underwent immunohistochemistry staining for Aβ (red), nuclei (blue), and either GFAP for astrocytes/Müller cells (green) or IBA-1 for microglia (green) or TUBB3 for neuronal microtubules (green). Aβ immunofluorescence was evident within what is likely the retinal ganglion cells (**asterisk**) and the neuropil and extracellular spaces (**arrowheads**). Double labelling is seen (**arrows**). Stronger Aβ immunoreactivity (red) was observed intracellularly in retinal ganglion cells and in the neuropil of AD donors compared to controls. Scale bar = 25 µm

Figure 7 demonstrates representative cross-sections after double labelling immunoreactivity for Aβ/GFAP, Aβ/IBA-1 and Aβ/TUBB3. Aβ immunoreactivity (red) was present in what was likely the cytoplasmic compartment of some retinal ganglion cells (asterisks and inserts) and the extracellular spaces within the neuropil (arrowheads) in AD and control eyes. Likely extracellular deposits (arrowheads) are shown as clusters or single specks of Aβ immunoreactivity. Double labelled deposits are noted by arrows. Note that there was more Aβ labelling in the AD eyes, consistent with the quantitative analysis shown in Fig. 3.

Glial cells in the retina were sampled using antibodies against GFAP and IBA-1. GFAP is a 50 kDa filamentous cytoskeletal protein found in retinal astrocytes and Müller cells. Few double labelled profiles were observed in either the AD or control retinal sections immunoreacted for Aβ and GFAP (Fig. 7 A, B). GFAP labelling was present predominantly in the RNFL and GCL, and more abundant in the control eyes compared to AD eyes, consistent with the quantitative analysis in Fig. 4A.

**Fig. 8:**
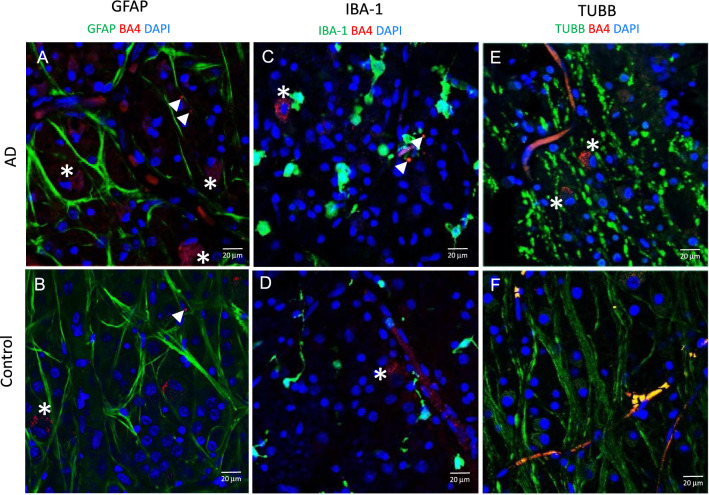
Representative immunofluorescence images of retinal punches. **A**, **C**, **E** Post-mortem human retina samples were processed from AD donors (Mean age = 75.3) (N = 3). **B**, **D**, **F** Post-mortem human retina samples were processed from controls (Mean age = 70.7) (N = 3). Tissues underwent immuno-histochemistry staining for Aβ (red), and either GFAP for astrocytes/Müller cells (green), IBA-1 for microglia (green) or TUBB3 for neuronal microtubules (green). Aβ (red) immunofluorescence was evident within what was likely the retinal ganglion cells **(asterisk)** and in the neuropil and extracellular spaces **(arrows)**. Stronger Aβ immunoreactivity (red) was observed intracellularly in retinal ganglion cells and in the neuropil of AD donors compared to controls. Note abnormal nodular appearance of axonal profiles (green TUBB3) in AD (**E**). Scale bar = 20 µm. Error bars = Standard Error

IBA-1 is a 17 kDa protein that is specifically expressed in the microglia/macrophage lineage in the retina. IBA-1 immunoreactivity was mostly observed in microglial processes in the inner layers of both AD and control eyes (Fig. 7 C, D). The distribution of IBA-1 was significantly higher in the mid-peripheral (~ 10 mm from ONH) compared to the central retina (~ 5 mm from ONH) in the AD eyes only, suggesting a greater microglial response to the higher levels of Aβ in the mid-peripheral (~ 10 mm from ONH) retina of the AD eye (Fig. 4B). Interestingly, even though levels of IBA-1 and Aβ were generally lower in the control eyes, the control eyes had greater co-localization of IBA-1 and Aβ compared to AD eyes (Fig. 4B, yellow profiles in Figs. 7D, and 8).

Sections processed for immunoreactivity against Aβ and TUBB3, demonstrate similar patterns of labelling in the AD (Fig. 7E) and control (Fig. 7F) eyes including double labelled (yellow/orange) retinal ganglion cells, as well as single (green) and double labelled (yellow/orange) axonal profiles.

In addition to the retinal cross-sections, we also studied the immunolabelling in the horizontal plane in wholemount retinal punches (Fig. 8). The GFAP immunolabelling (Fig. 8A, B) revealed morphologies of macroglial processes in the AD and control eyes. IBA-1 immunolabelling (Fig. 8C, D) revealed more amoeboid microglial cell profiles in the AD compared to the control eyes. The increased surface area of amoeboid microglia is consistent with higher levels of IBA-1 immunofluorescence in AD (Fig. 4). Control eyes demonstrated microglial processes that were more reminiscent of the resting state of microglia. TUBB3 immunolabelling (Fig. 8E, F) revealed a unique nodular and tortuous morphology of the axonal processes in the AD eye, which may be an anatomical correlate associated with axonal degeneration of the retinal ganglion cells.

Additional file 1: Figure S1 shows Aβ labelling in z-stack image volumes of AD and control retinal punches, scanning through the layers from RNFL to INL. Additional file 1: Figure S2 shows additional examples of GS and GFAP double-labelling at higher power. Additional file 1: Figure S3 shows quantitative methods used to analyse macroglia. Additional file 1: Figure S4 illustrates double labelling.

### Aβ co-localized with neuronal profiles in both control and AD eyes, Aβ co-localized with microglia predominantly in control eyes

**Fig. 9:**
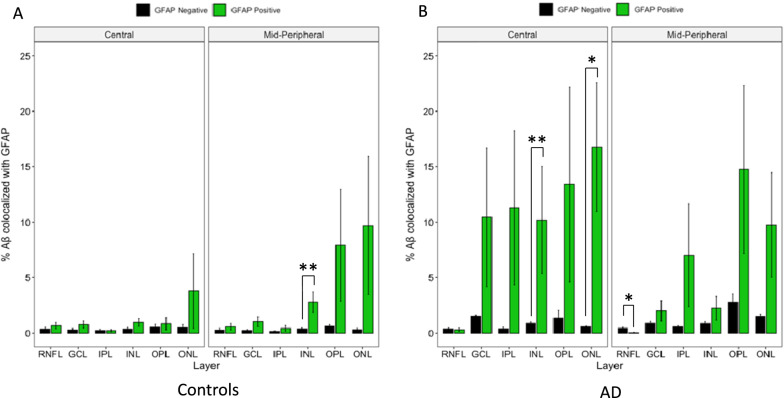
Layer-wise cross-sectional colocalization profile of GFAP labelled astrocytes with Aβ. Left panel illustrate age-matched controls (N = 7). Right panel illustrate AD donors (N = 5). The primary antibody against GFAP was visualized using a FITC-labelled secondary antibody. The primary antibody against Aβ was visualized using a Cy3-labelled secondary antibody. Green bars represent the percentage of Aβ positive pixels among FITC (GFAP) -positive pixels. Black bars represent the percentage of Aβ positive pixels among FITC (GFAP) -negative pixels. Colocalization is defined by FITC-positive pixels having a significantly higher percentage of Aβ positivity than FITC-negative pixels. Aβ colocalization with GFAP is calculated in each retinal layer and plotted on the x-axis. One out of 12 geo-layers in controls (**A**) and 3 out of 12 geo-layers in AD donors (**B**) showed significant Aβ-GFAP colocalization. * represent *p* < 0.05. ** represent *p* < 0.01. Error bars = Standard Error

**Fig. 10:**
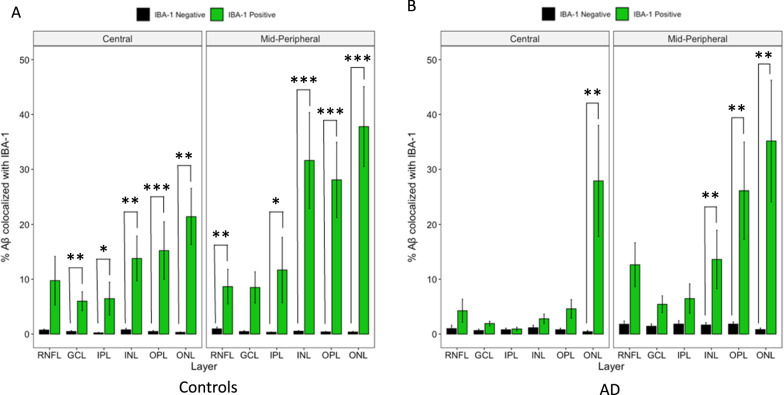
Layer-wise cross-sectional colocalization profile of IBA-1 labelled microglia with Aβ. Left panel illustrate age-matched controls (N = 7). Right panel illustrate AD donors (N = 5). The primary antibody IBA-1 was visualized using a FITC-labelled secondary antibody. The primary antibody against Aβ was visualized using a Cy3-labelled secondary antibody. Green bars represent the percentage of Aβ positive pixels among FITC (IBA-1) -positive pixels. Black bars represent the percentage of Aβ positive pixels among FITC (IBA-1) -negative pixels. Colocalization is defined by FITC (IBA-1) -positive pixels having a significantly higher percentage of Aβ positivity than FITC-negative pixels. Aβ colocalization with IBA-1 is calculated in each retinal layer and plotted on the x-axis. Ten out of 12 geo-layers in controls (**A**) and 4 out of 12 geo-layers in AD donors (**B**) showed significant Aβ-IBA-1 colocalization. * represent *p* < 0.05. **represent *p* < 0.01. *** represent *p *< 0.001. Error bars = Standard Error

**Fig. 11:**
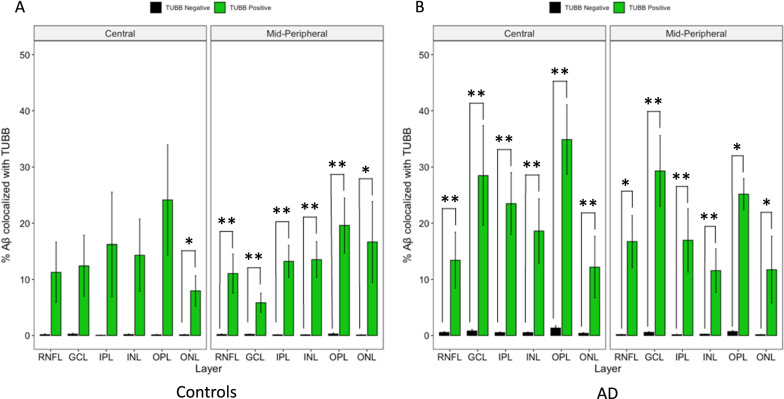
Layer-wise cross-sectional colocalization profile of TUBB3 labelled neurons with Aβ. Left panel illustrate age-matched controls (N = 7). Right panel illustrate AD donors (N = 5). The primary antibody against TUBB3 was visualized using a FITC-labelled secondary antibody. The primary antibody against Aβ was visualized using a Cy3-labelled secondary antibody. Green bars represent the percentage of Aβ positive pixels among FITC (TUBB3) -positive pixels. Black bars represent the percentage of Aβ positive pixels among FITC (TUBB3) -negative pixels. Colocalization is defined by FITC (TUBB3) -positive pixels having a significantly higher percentage of Aβ positivity than FITC-negative pixels. Each bar-pair represent a geo-layer. Aβ colocalization with TUBB3 is calculated in each retinal layer and plotted on the X-axis. Seven out of 12 geo-layers in controls (**A**) and 12 out of 12 geo-layers in AD (**B**) showed significant Aβ-TUBB3 colocalization. *represent *p *< 0.05. **represent *p*  < 0.01. Error bars = Standard Error

Figures 9, 10 and 11 display Aβ co-localization analysis with each of the three cell markers: GFAP labelled astrocytes, IBA-1 labelled microglia, and TUBB3 labelled neurons, respectively. All three markers were visualized using FITC secondary antibody fluorescence in separate experimental cross-section slide preparations. Green bars represent the percentage of Aβ-positive pixels among FITC-positive pixels, while the black bars represent the percentage of Aβ-positive pixels among FITC-negative pixels. If Aβ immunolabelling occurred on all locations (pixels) randomly, there would be no statistically significant difference between these percentages (green and black bars). Instead, we saw a significantly larger percentage of FITC-positive pixels that were also labelled with Aβ, indicating that Aβ immunolabelling preferentially co-localized with each cell marker in their respective cohort.

The co-localization results were analyzed in geo-layers, defined as categories representing a specific geographical region (ie. Central (~ 5 mm from ONH) or mid-peripheral (~ 10 mm from ONH)) and a specific retinal layer (ie. RNFL, GCL, etc.). Geo-layers containing Aβ/GFAP co-localization were compared between AD and control eyes (Fig. 9A and B). In total, only 4 out of 24 geo-layers reached significance, namely control mid-peripheral (~ 10 mm from ONH) INL (*p* < 0.01), AD central (~ 5 mm from ONH) INL (*p* < 0.01), AD central ONL (*p* < 0.05), and AD mid-peripheral RNFL (*p* < 0.05), indicating Aβ/GFAP co-localization was less prevalent than Aβ/TUBB3, described later. Figure 9 shows 5.18% of AD astrocytes are co-localized with Aβ, and 0.96% of control astrocytes are co-localized with Aβ. As macroglia mainly function to maintain neuronal homeostasis and are not known to play a phagocytic role, as with microglia, co-localization with Aβ is not generally expected of macroglia cells.

Next, we assessed the Aβ/IBA-1 co-localization between AD and control eyes (Fig. 10 A and B). There was significant Aβ/IBA-1 co-localization in 10 out of 12 geo-layers in control eyes. However, in AD eyes, there was significant Aβ/IBA-1 co-localization in only 4 out of 12 geo-layers. Figure 10 shows 8.86% of control microglia is co-localized with Aβ and 4.60% of AD microglia is co-localized with Aβ. This was a novel finding, one that may suggest that the phagocytic role of microglia is dysfunctional in AD eyes.

Figure 11 A and B displays the layer-wise analysis of Aβ co-localization with TUBB3-labelled neurons in AD and control eyes. There was significant Aβ/TUBB3 co-localization in all mid-peripheral retinal layers (~ 10 mm from ONH) of both controls and AD donors (*p* < 0.05). In the central retina (~ 5 mm from ONH), there was significant Aβ/TUBB3 co-localization in all layers of the AD donors (*p* < 0.01), but only in the ONL of the control eyes (*p* < 0.05). This is consistent with (and possibly due to) the fact that there is a lower amount of Aβ in control eyes compared to AD eyes, which is further compounded by the finding that central retina (~ 5 mm from ONH) has even less Aβ compared to mid-peripheral retina (~ 10 mm from ONH) in both control and AD eyes [42]. Nineteen out of 24 geo-layers of the combined AD and control eyes showed significant co-localization between Aβ and TUBB3 (Fig. 11 A and B). This is consistent with the literature since it is known that Aβ is a cleaved product of amyloid precursor protein (APP), a protein that is produced principally by neurons in the CNS. Figure 11 shows 14.85% of AD neurons are co-localized with Aβ, and 5.68% of control neurons are co-localized with Aβ.

#### Aβ colocalization by microglia compared between control and AD retina

**Fig. 12:**
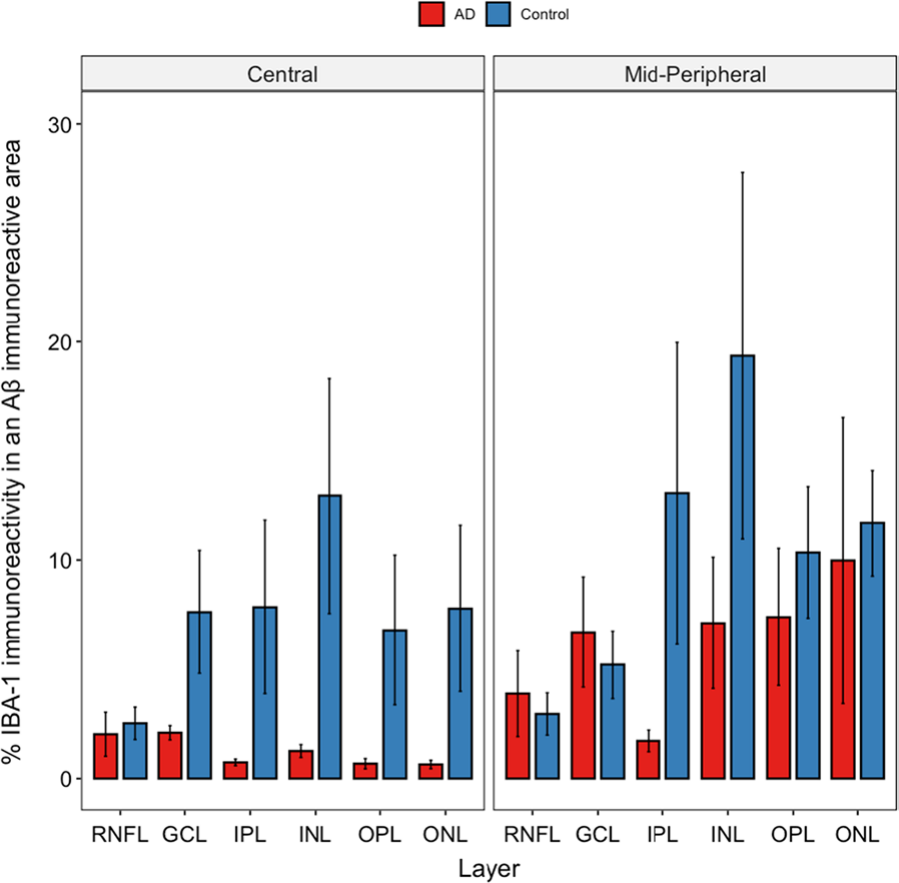
Aβ co-localization with Microglia. Percent of IBA-1 immunoreactivity in a BA4 immunoreactive area was plotted against the retinal layers in central vs. mid-peripheral retina. Red bars represent AD donors (N = 5). Blue bars represent age-matched controls (N = 7). Although the average values of IBA-1 immunoreactivity in a BA4 immunoreactive area in the control eyes were larger than those in the AD eyes, the difference did not reach significance (alpha = 0.05), Error bars = Standard Error.

In the retinal tissues, microglia are one of the cell types capable of phagocytic function needed to destroy abnormal or toxic deposits, such as Aβ. Therefore, we studied the percentage of IBA-1 labelling that was co-localized with Aβ to further assess the relationship between microglia and Aβ deposits, as this would shed light on the ability of microglia to phagocytose Aβ. Figure 12 shows the result of this analysis. The x-axis shows retinal layers in the central (~ 5 mm from ONH) and mid-peripheral retina (~ 10 mm from ONH), while the Y-axis shows the percentage of Aβ-labelled pixels that were co-localized by microglia. The difference between the control and the AD eyes in Aβ-colocalization by microglia was not significant but the geo-layer averages were larger for the control eyes in 10 out of 12 geo-layers. We note that although not significant, this result may be interpreted with the co-localization result in Fig. 10 which compares the Aβ concentration in the microglia regions vs. non-microglia regions, to test if Aβ indeed is preferentially co-localizing in the microglia-positive pixels. In Fig. 10, Aβ in the control eyes showed a strong preference for co-localizing in the microglia region compared to the non-microglia region, whereas this was less consistent in the AD eyes. A similar test in Fig. 11 showed that Aβ in the AD retina was preferentially co-localized in the neuronal profile region, compared to the non-neuronal region across all regions and layers, and this was less consistently so in the control retina in the central region.

## Discussion

### Higher Aβ load in AD compared to control retina

Our results demonstrated Aβ levels were significantly higher in the mid-peripheral retina (~ 10 mm from ONH) of AD compared to controls. For central retina (~ 5 mm from ONH), we observed Aβ levels to be higher in AD compared to controls, but this did not reach significance (Fig. 3). Our findings are in agreement with our earlier study [42] and those of other labs [37, 38]. Lee et al. showed a higher amount of Aβ in mid-peripheral retina of AD donors, with no significant difference between AD and controls in the central retina [42]. Koronyo et al. found retinal Aβ in the mid- and far-periphery in both AD donors and live AD patients [37]. Many factors may have contributed to the topographical difference in Aβ deposition, including retinal blood vessel parameters, tissue permeability and light stimulation [37]. The mid-peripheral retina was also shown to have more intermediate hard drusen in AD eyes compared to controls, which is consistent with the theory that physiological differences between topographical areas may provide a permissive environment for abnormal deposition to occur in the mid- and far-peripheral retina [13, 83]. 

Our quantitative data were obtained from immunohistochemical processing of human eye tissues with the BA4 antibody. BA4 labels Aβ but also may additionally label full-length APP [49]. However, we also used 12F4, an antibody that is considered specific for Aβ and compared labelling patterns with BA4. In Fig. 2, we show that both BA4 and 12F4 have similar labelling patterns, including the presence of intraneuronal Aβ in retinal ganglion cells (See Table 1 in [49]). However, in de Haan et al. 2018, 12F4 and an APP-specific antibody co-localized to retinal ganglion cells, suggesting the possibility that even 12F4 may not show specificity to the cleaved Aβ peptide [18]. Of note, the full-length protein, APP, should be present in all retinal ganglion cells, yet our immunolabeling results demonstrated that Aβ/BA4 and Aβ/12F4 labelled only a very small minority of retinal ganglion cells in our study. Future studies should focus on the development of more specific antibodies which can be assayed in western blot (WB) methods that can distinguish full-length APP (~ 100 kDa) from cleaved Aβ peptide (~ 4 kDa) by WB banding patterns and their relative molecular weights.

Although many studies support the presence of increased Aβ in the AD retina, some controversy remains in the literature. Qualitative studies by den Haan et al. did not see a difference in Aβ deposition in the AD retina compared to controls [18]. They reported that Aβ and tau deposits were present in the retina, although they do not resemble the typical pattern seen in the brain. Our findings on retinal Aβ concur, as likely extracellular Aβ appears as small, punctate deposits, unlike the brain Aβ plaques, which are several times larger than retinal Aβ deposits. Williams et al., reported that they were unable to find immunohistochemical evidence for Aβ deposits in any part of the globe [86].

In this study and our earlier study, we observed an increased number of retinal ganglion cells that contained likely intracellular Aβ in the AD retina compared to controls [42]. In the AD brain, intracellular Aβ plays a key role in early neuronal and synaptic dysfunction. Intracellular Aβ is observed prior to extracellular Aβ deposition, suggesting its potential as an earlier indicator of AD pathology [24, 79]. If intracellular Aβ precedes extracellular Aβ deposition in the retina, as it does in the brain, our results may indicate that the AD retina lags behind the AD brain in progression of extracellular deposits of Aβ [24, 79]. This may be due to extracellular retinal deposits of Aβ being more readily cleared by the retinal vasculature than in the brain. Future studies are needed to further understand the different morphologies of Aβ deposits in the retina compared to the brain.

### Metabolic decline of Müller cells in AD retina

Our initial GFAP immunoreactivity results suggested degeneration of GFAP labelled macroglia in the post-mortem human retina, which seemed at odds with reports of increased GFAP immunoreactivity in AD brain tissues compared to controls [60, 85]. Unique to the retina, GFAP also labels Müller cells, a specialized type of macroglia not present in brain tissues. As GFAP can label both astrocytes and Müller cells, GS was introduced as a Müller-specific cell marker. Within the macroglia cells, activated Müller cells, identified by having both GS and GFAP immunoreactivity, displayed lower levels in AD compared to the control retina (Fig. 6A). It is known that declining GS activity can indicate progression of neurodegeneration in many neurological disorders, potentially due to glutamate toxicity [35]. However, we were unable distinguish whether the loss of GS immunoreactivity was due to a decrease in the number of Müller cells, or merely a decline in the GS enzyme within the Müller cells. Both conditions would result in lowered glutamine synthesis and subsequent imbalances in GABA and glutamate neurotransmitters.

While there are no studies, to the best of our knowledge, that directly compares GS changes in the AD eye and brain, previous studies have shown conflicting evidence of GS levels in the AD brain. Most studies showed a decrease in GS activity and levels in AD, including in the post-mortem human brain and transgenic mouse models [7, 39, 41, 58, 67, 68, 80]. Other studies showed an increase of GS in the prefrontal cortex and cerebrospinal fluid [6, 82]. GS in the brain mainly exist in astrocytes, while GS in the retina mainly exist in Müller cells [22, 35]. Therefore, elevated GS in the brain may represent part of the astrogliotic process, whereas decreased GS in the retina may represent Müller cell degeneration. Interestingly, structural perturbation of the GS protein was seen in the AD brain, as well as with exposure of GS protein to Aβ [7]. This raises the possibility of GS dysfunction in an environment rich with Aβ, which is confirmed by a later study that showed lower GS activity and higher GS level in the AD brain [10]. In light of these studies, it seems probable that GS plays a unique, and likely different, roles in the retina compared to the brain.

### Microgliosis

As part of embryonic and postnatal development, microglia enter the retina as mononuclear phagocytes via the ciliary body and hyaloid. Microglia are known to be located in the GCL, IPL and OPL [64], although they can migrate to any layer of the retina in response to apoptotic cells or toxic deposits such as Aβ [84].

Our study revealed higher amounts of IBA-1-labelled microglia in AD donor eyes compared to controls (Fig. 4B), which is consistent with the literature, as microglial activation and microgliosis in neurodegenerative diseases has been well documented [55, 62, 69]. Microglia activation was significantly higher in the retina of a mouse model of AD [61]. In a similar experiment performed by Grimaldi and colleagues, a higher number and density of IBA-1-positive cells were seen in AD donor retina compared to controls [25]. The increased microglial activation in AD (and associated higher levels of Aβ) is consistent with the pro-inflammatory microglia hypothesis, which states that chronic microglia activation due to Aβ deposition leads to neurotoxicity [1, 73]. Interestingly, although AD pathogenesis may lead to more microglia activation, the quality of the microglial response may not be as robust. In our study, Aβ/IBA-1 co-localization was less consistent throughout layers in AD eyes compared to controls (Fig. 10), suggesting microglial cell dysfunction. In addition, fewer retinal layers in AD eyes showed significant Aβ/IBA-1 co-localization than controls (Fig. 10). The microglial dysfunction hypothesis proposes that the ability of microglial cells to phagocytize and clear Aβ is blunted and reduced due to Aβ accumulation in AD, and thereby plays a role in AD pathogenesis [78]. Microglial dysfunction may include inappropriate microglial activation and the loss of its neurosupportive function [55, 75–77].

To further explore microglial dysfunction in AD donors, we analyzed the percentage of IBA-1 immunoreactivity that co-localized with Aβ. This indirectly relates to how robustly microglia migrate towards or phagocytize Aβ (Fig. 12) [15]. Consistent with Aβ co-localization data (Fig. 10), control retina had more IBA-1 immunoreactivity co-localized with Aβ compared to AD retina, suggesting that microglia in control eyes maintained their immunological function to migrate towards and phagocytize Aβ better than in AD eyes (Fig. 12). These data are consistent with the proliferation of dysfunctional microglia, with the exhaustion of healthy microglial cells, observed in the brain [15, 28, 45, 55].

**Fig. 13:**
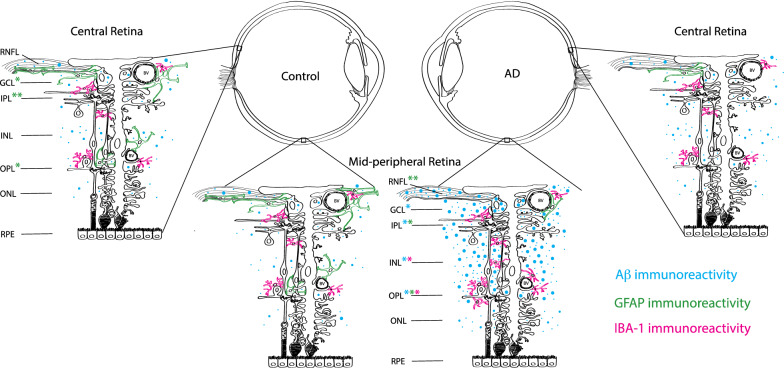
Summary schematic of layer-wise location of Aβ, GFAP, and IBA-1 immunostaining. Representative schematic demonstrates the qualitative and quantitative data from this study. Blue represents Aβ immunoreactivity. Green represents GFAP immunoreactivity. Pink represents IBA-1 immunoreactivity. AD retina is shown on the right, and control retina is shown on the left. The asterisks on the layer abbreviations represents the types of cells seen in this layer. *RNFL* Retinal nerve fiber layer. *GCL* Ganglion cell layer. *IPL* Inner plexiform layer. *INL* Inner nuclear layer. *OPL *Outer plexiform layer. *ONL *Outer nuclear layer. *RPE* Retinal pigment epithelium. *Represent *p* < 0.05. **Represent *p* < 0.01

Figure 13 shows a summary schematic of layer-wise location of Aβ, GFAP, and IBA-1 immunostaining. The representative schematic demonstrates the qualitative and quantitative data from this study. Blue represents Aβ immunoreactivity. Green represents GFAP immunoreactivity. Pink represents IBA-1 immunoreactivity. AD retina is shown on the right, and control retina is shown on the left. The asterisks on the layer abbreviations represent the types of cells seen in this layer.

## Conclusion

### New strategies in biomarkers for AD

This study is the first to analyze layer-wise Aβ distribution in a quantitative manner and in relationship to glial profiles in the postmortem human retina. In vivo imaging of retinal Aβ with fluoroprobes has made tremendous progress for staging and diagnosing individuals with AD [17, 37, 38, 74]. In addition, hyperspectral retinal imaging may also provide information on retinal Aβ as an additional tool for non-invasive Aβ detection [8, 26]. Our study demonstrated that both Muller degeneration and microgliosis may be promising biomarkers in addition to Aβ. In fact, in the occipital lobe, microglia activation was quantified in vivo with the (R)-[11C]PK11195 positron emission tomography (PET) ligand, which was significantly increased in AD patients compared to healthy controls [72]. In the eye, a recent study reported in vivo hyperreflective granular membranes consisting of microgliosis material, visualized on adaptive optics scanning laser ophthalmoscopy, raising the possibility of using microgliosis as a new potential biomarker for early AD detection [90]. Fewer studies have identified in vivo probes for astrocyte and. future studies are needed to develop novel astrocyte probes for in vivo retinal imaging, as well as to characterize Müller degeneration at early stages AD in the retina [19].

## Supplementary Information

**Additional file 1**. Supplementary Figures. **Figure S1** shows Aβ labelling in z-stack image volumes of AD and control retinal punches, scanning through the layers from RNFL to INL; **Figure S2** shows additional examples of GS and GFAP double-labelling at higher power; **Figure S3** shows quantitative methods used to analyse macroglia; **Figure S4** illustrates double labelling.

**Additional file 2**. All p-values.

## Data Availability

Data and material for the study are stored at the University of British Columbia. Additional data and material are available upon request.

## Abbreviations

AD: Alzheimer’s disease
Aβ: Amyloid-β protein
pTau: Hyper-phosphorylated tau proteins
MRI: Magnetic resonance imaging
PET: Positron emission tomography
TUBB3: Tubulin-β3
GFAP: Glial fibrillary acidic protein
IBA-1: Ionized calcium binding adapter molecule-1
PBS: Phosphate buffered saline
OCT: Optical coherence tomography
ONH: Optic nerve head
