# Supplementary material for: Müller cell degeneration and microglial dysfunction in the Alzheimer’s retina
Source: Acta Neuropathol Commun. 2022 Oct 5;10:145. doi: 10.1186/s40478-022-01448-y (PMC9533552; doi:10.1186/s40478-022-01448-y)
Supplement: Supplementary file 1 — Additional file 1. Supplementary Figures. Figure S1 shows Aβ labelling in z-stack image volumes of AD and control retinal punches, scanning through the layers from RNFL to INL; Figure S2 shows additional examples of GS and GFAP double-labelling at higher power; Figure S3 shows quantitative methods used to analyse macroglia; Figure S4 illustrates double labelling. [file 40478_2022_1448_MOESM1_ESM.pptx]

## Slide 1
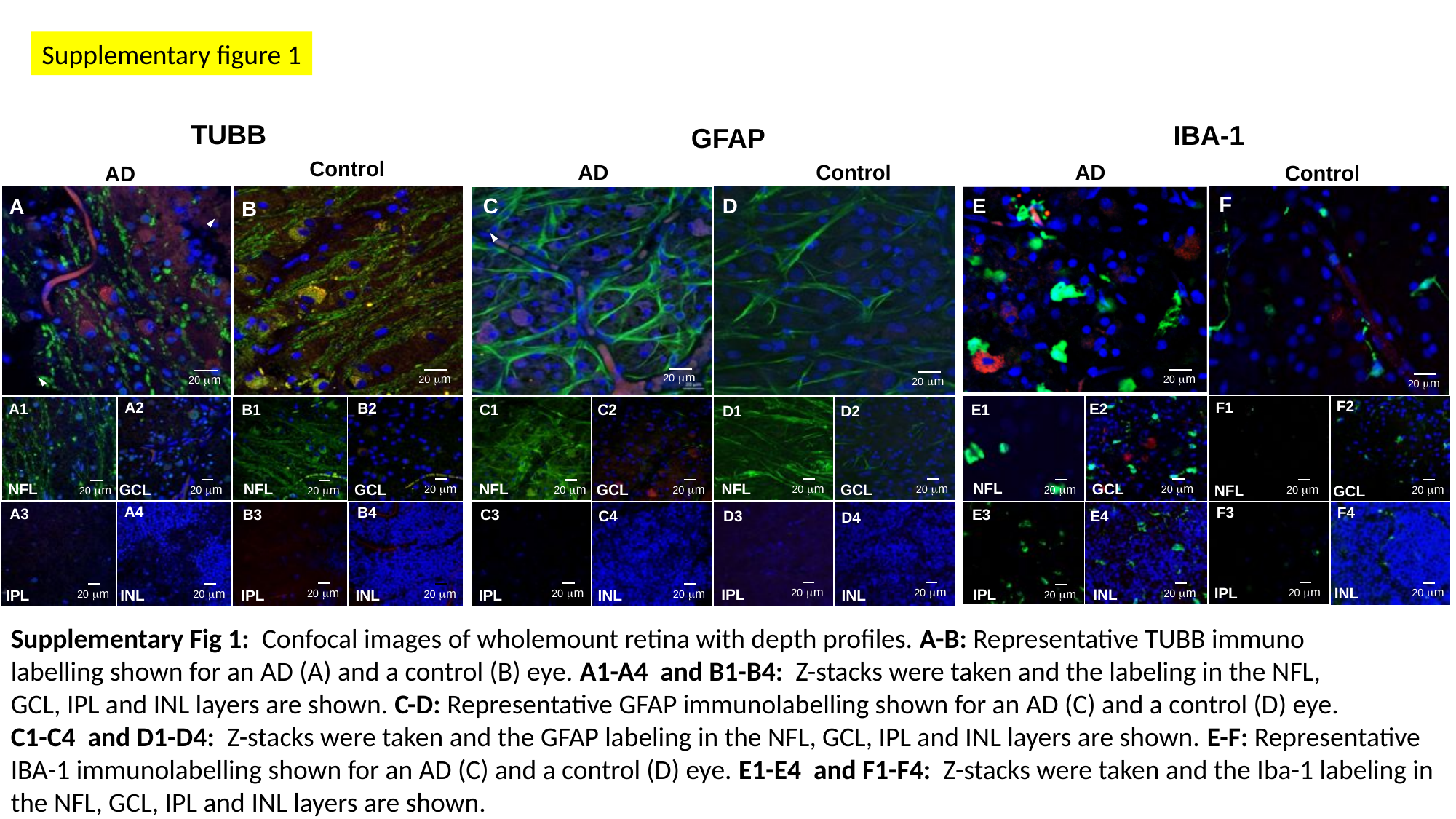

Supplementary figure 1
TUBB
IBA-1
GFAP
Control
AD
Control
AD
Control
AD
F
E
C
D
A
4
B
20 mm
20 mm
20 mm
20 mm
2015-0471T
18-41I
VA12-38I
20 mm
2016-00245I
2012-193I
VA12-64T
20 mm
F2
F1
A2
B2
A1
E2
E1
C2
B1
C1
D2
D1
NFL
NFL
GCL
NFL
NFL
NFL
GCL
GCL
GCL
GCL
20 mm
NFL
20 mm
20 mm
GCL
20 mm
20 mm
20 mm
20 mm
20 mm
20 mm
20 mm
20 mm
20 mm
A4
F3
B4
F4
A3
E3
B3
C3
E4
D3
C4
D4
IPL
INL
20 mm
20 mm
20 mm
IPL
20 mm
20 mm
20 mm
20 mm
IPL
INL
IPL
IPL
IPL
INL
20 mm
20 mm
INL
INL
INL
20 mm
20 mm
20 mm
Supplementary Fig 1: Confocal images of wholemount retina with depth profiles. A-B: Representative TUBB immuno
labelling shown for an AD (A) and a control (B) eye. A1-A4 and B1-B4: Z-stacks were taken and the labeling in the NFL,
GCL, IPL and INL layers are shown. C-D: Representative GFAP immunolabelling shown for an AD (C) and a control (D) eye.
C1-C4 and D1-D4: Z-stacks were taken and the GFAP labeling in the NFL, GCL, IPL and INL layers are shown. E-F: Representative IBA-1 immunolabelling shown for an AD (C) and a control (D) eye. E1-E4 and F1-F4: Z-stacks were taken and the Iba-1 labeling in the NFL, GCL, IPL and INL layers are shown.

## Slide 2
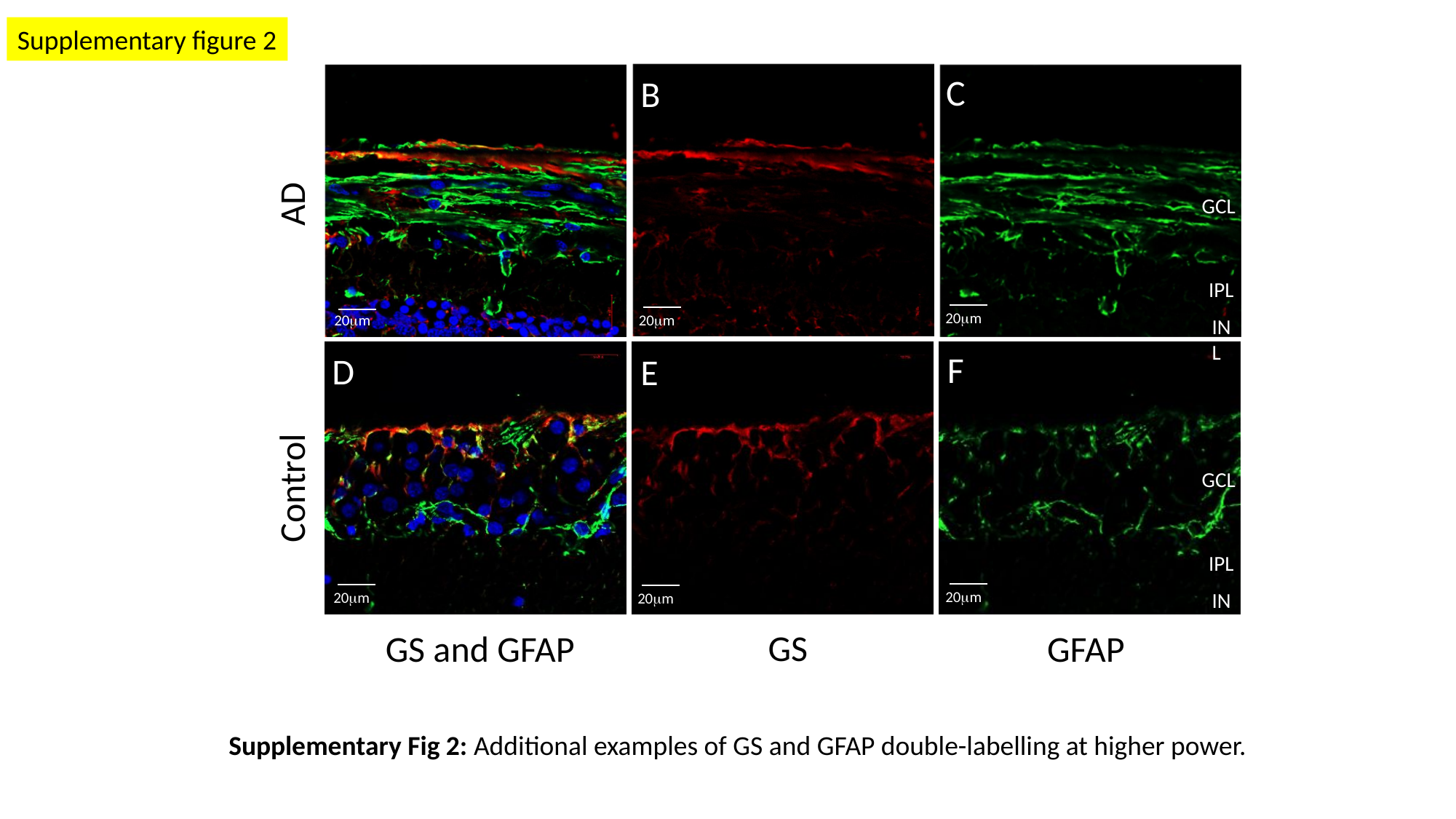

Supplementary figure 2
C
A
B
AD
GCL
IPL
20mm
20mm
20mm
INL
F
D
E
GCL
Control
IPL
20mm
INL
20mm
20mm
GS
GS and GFAP
GFAP
Supplementary Fig 2: Additional examples of GS and GFAP double-labelling at higher power.

## Slide 3
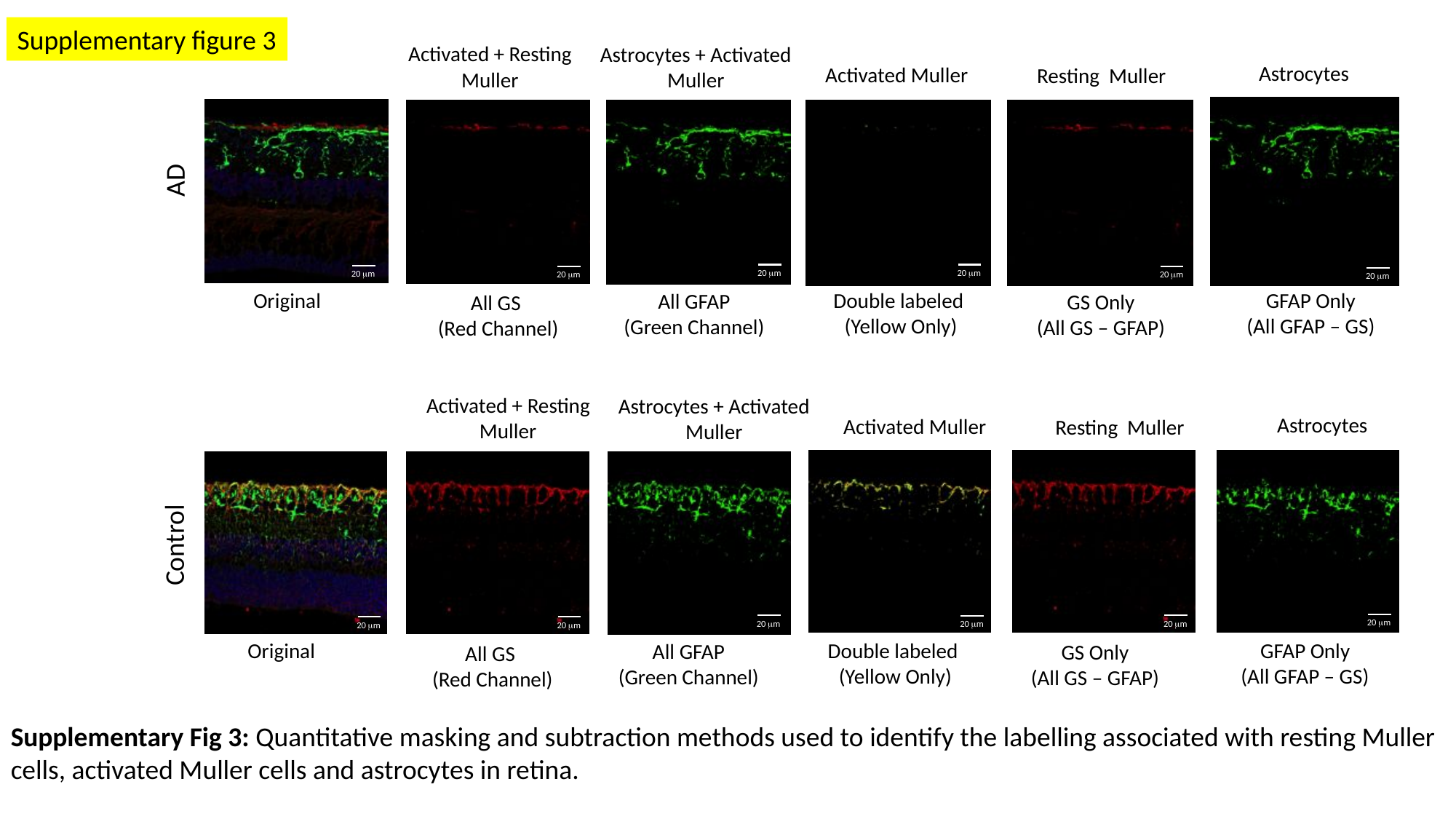

Supplementary figure 3
Activated + Resting
Muller
Astrocytes + Activated
Muller
Astrocytes
Activated Muller
Resting Muller
AD
20 mm
20 mm
20 mm
20 mm
20 mm
20 mm
Original
Double labeled
(Yellow Only)
GFAP Only
(All GFAP – GS)
All GFAP
(Green Channel)
GS Only
(All GS – GFAP)
All GS
(Red Channel)
Activated + Resting
Muller
Astrocytes + Activated
Muller
Astrocytes
Activated Muller
Resting Muller
Control
20 mm
20 mm
20 mm
20 mm
20 mm
20 mm
Original
Double labeled
(Yellow Only)
GFAP Only
(All GFAP – GS)
All GFAP
(Green Channel)
GS Only
(All GS – GFAP)
All GS
(Red Channel)
Supplementary Fig 3: Quantitative masking and subtraction methods used to identify the labelling associated with resting Muller cells, activated Muller cells and astrocytes in retina.

## Slide 4
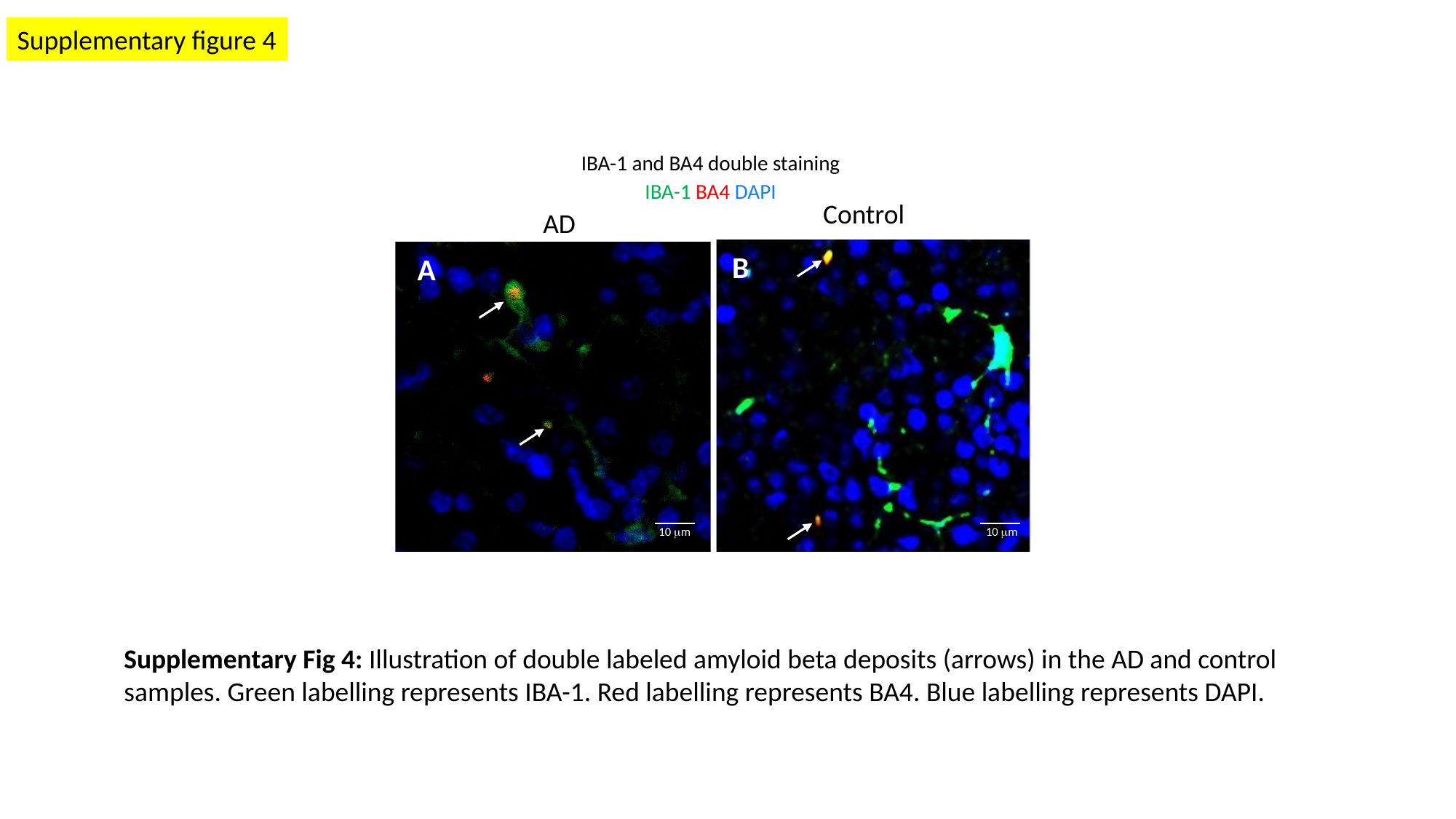

Supplementary figure 4
IBA-1 and BA4 double staining
IBA-1 BA4 DAPI
Control
AD
B
A
10 mm
10 mm
Supplementary Fig 4: Illustration of double labeled amyloid beta deposits (arrows) in the AD and control samples. Green labelling represents IBA-1. Red labelling represents BA4. Blue labelling represents DAPI.
